# Encapsulin Based Self-Assembling Iron-Containing Protein Nanoparticles for Stem Cells MRI Visualization

**DOI:** 10.3390/ijms222212275

**Published:** 2021-11-12

**Authors:** Anna N. Gabashvili, Stepan S. Vodopyanov, Nelly S. Chmelyuk, Viktoria A. Sarkisova, Konstantin A. Fedotov, Maria V. Efremova, Maxim A. Abakumov

**Affiliations:** 1 Department of Medical Nanobiotechnology, Pirogov Russian National Research Medical University, Ostrovityanova St., 1, 117997 Moscow, Russia; gabashvili.anna@gmail.com (A.N.G.); nellichmelyuk@yandex.ru (N.S.C.); cfvallwork@gmail.com (K.A.F.); 2 Laboratory “Biomedical Nanomaterials”, National University of Science and Technology “MISiS”, Leninskiy Prospect, 4, 119049 Moscow, Russia; stepan.vodopianov@yandex.ru; 3 Biological Faculty, Lomonosov Moscow State University, Leninskiy Gory, 1, 119234 Moscow, Russia; alice-lyddell@yandex.ru; 4 Engelhardt Institute of Molecular Biology, Vavilova Street, 32, 119991 Moscow, Russia; 5 Department of Chemistry and TUM School of Medicine, Technical University of Munich, Ismaninger Str. 22, 81675 Munich, Germany; mariia.efremova@helmholtz-muenchen.de; 6 Institute for Synthetic Biomedicine, Helmholtz Zentrum München GmbH, Ingolstaedter Landstr. 1, 85764 Oberschleissheim, Germany

**Keywords:** encapsulins, magnetic resonance imaging, cell tracking

## Abstract

Over the past decade, cell therapy has found many applications in the treatment of different diseases. Some of the cells already used in clinical practice include stem cells and CAR-T cells. Compared with traditional drugs, living cells are much more complicated systems that must be strictly controlled to avoid undesirable migration, differentiation, or proliferation. One of the approaches used to prevent such side effects involves monitoring cell distribution in the human body by any noninvasive technique, such as magnetic resonance imaging (MRI). Long-term tracking of stem cells with artificial magnetic labels, such as magnetic nanoparticles, is quite problematic because such labels can affect the metabolic process and cell viability. Additionally, the concentration of exogenous labels will decrease during cell division, leading to a corresponding decrease in signal intensity. In the current work, we present a new type of genetically encoded label based on encapsulin from *Myxococcus xanthus* bacteria, stably expressed in human mesenchymal stem cells (MSCs) and coexpressed with ferroxidase as a cargo protein for nanoparticles’ synthesis inside encapsulin shells. mZip14 protein was expressed for the enhancement of iron transport into the cell. Together, these three proteins led to the synthesis of iron-containing nanoparticles in mesenchymal stem cells—without affecting cell viability—and increased contrast properties of MSCs in MRI.

## 1. Introduction

While using cells for therapy or establishing preclinical models, it is critically important to maintain the possibility of long-term cell tracking after administration into the organism. For example, in some cancer models, the formation of bone metastasis can take up to a few weeks or even months [1]. The migration processes of neural stem cells in tissues after transplantation can take approximately the same time [2]. The labels used for such long-term tracking should have minimal effects on the processes of cell division, migration, and proliferation—and should not change cell viability.

Nowadays, many different approaches are used to create genetically encoded labels in cells for their monitoring after administration into the body. One approach is fluorescent optical monitoring using quantum dots and fluorophores [3,4,5] or gene-encoding fluorescent proteins [6,7] as labels. The main disadvantage of these labels is their limited depth of detection (usually not more than 2 mm); this approach is only possible in surface tissues of small animals such as mice. Bioluminescent imaging is another viable alternative based on the reaction between the enzyme luciferase and its substrate luciferin. Firefly luciferase (Fluc), Renilla luciferase (Rluc), and bacterial luciferases [8] have been utilized for this purpose. The bioluminescent approach is more sensitive than the fluorescent approach, but the depth of signal penetration through the tissues is still limited to a few centimeters.

Other noninvasive techniques include SPECT (single-photon emission computed tomography) or PET (positron emission tomography), which utilize radioactive isotopes such as ^99^mTc, ^111^In, ^18^F, ^64^Cu [9,10,11,12]. However, the signal intensity of these labels will decrease over time due to the radioactive decay of isotopes. Moreover, radioactive labels can be excreted from cells, creating false-positive signals. The three main systems of reporter genes used for SPECT and PET include mammal receptors (e.g., dopamine receptor), mitochondrial proteins [13], and destabilized HSV1-tk protein [14]. Both SPECT and PET techniques demonstrate high sensitivity and spatial resolution compared to optical methods but require injections of radioactive compounds which are toxic to the patients [15].

The MRI technique is also utilized in cell monitoring. The most widespread labels used for MRI are magnetic nanoparticles (MNPs) with various chemical compositions, sizes, shapes, and surface properties [16,17,18]. For example, MNPs ranging in size from 16 to 200 nm produce mostly negative contrast due to their high T2 relaxation values, whereas small MNPs (less than 10 nm) demonstrate positive contrast properties, acting as a T1 contrast agent [19]. MNPs can be modified with various ligands (e.g., glucosamine) for increased biocompatibility and intracellular uptake [20]. Researchers use physical, chemical, and biological methods to achieve the desired shape, size, charge, and dispersion for maximum application efficiency of MNPs [21,22].

However, MRI is not free from disadvantages, similar to those of the SPECT and CT techniques. In particular, the signal intensity decreases over time because of cell division, while the probability of false-positive results increases due to MNP uptake by macrophages [23]. These challenges could be tackled by genetically encodable reporter systems for MRI; currently, these are primarily based on overexpression of intracellular metalloproteins such as transferrin receptors or ferritins. Transferrin receptor (TfR) can internalize iron ions bound to transferrin into the cell via receptor-mediated endocytosis [24]; an increase in expression of TfR leads to accumulation of intracellular iron detectable by MRI [17]. Ferritin is an iron storage protein that can accumulate up to 4000 iron atoms per molecule [25]. However, ferritin does not show remarkable magnetic properties and thus provides weak contrast in MRI.

In 1994, protein complexes with properties and structures similar to ferritin were discovered in *Brevibacterium linens* [26] and later in other bacteria [27,28]. Further studies have shown that these high-molecular-weight proteins are presented as capsid-like structures named encapsulins or bacterial nanocompartments. Bioinformatic analyses of different gene sequences have revealed thousands of such nanocompartments found in bacteria and archaea with a vast variety of cargo proteins [29,30]. Among nanocompartments [31], encapsulin from *Myxococcus xanthus (Mx),* first described in 2014, deserves special attention [32]. *Mx* encapsulin consists of a protein shell with ~32 nm diameter, encoded by the EncA gene, and cargo proteins, represented by EncB, EncC, and EncD. The protein shell of *Mx* encapsulin is self-assembled from 180 identical protomer proteins with a molecular weight equal to 32.5 kDa. Cargo proteins EncB (17 kDa), EncC (13 kDa), EncD (11 kDa) have ferritin-like domains attached to the inner surface of the encapsulin shell. Importantly, *Mx* encapsulin is shown to store up to 30,000 iron atoms [32], which makes it a conceivable candidate for genetically encoded intracellular labeling in MRI. Recently, the principal possibility of transient heterological expression of encapsulin-encoding genes in mammalian cells was demonstrated [33,34].

In our work, we present genetically encoded labels based on the *Mx* encapsulin system expressed in human adipose-derived mesenchymal stem cells (hAD-MSCs). Genes encoding encapsulin shell protomer (EncA-FLAG), cargo proteins with ferroxidase activity (EncBCD), and genes encoding transmembrane bivalent iron transporter (mZip14) (required for enhanced intracellular iron transfer) were inserted into MSCs genome via lentiviral transduction, allowing us to obtain a stable cell line. After adding ferrous ammonium sulfate (FAS), bivalent iron was transported into the cell by mZip14 and formed iron-containing crystals inside the encapsulin shell. Such genetically encoded labels in MSCs cells do not affect their proliferation and viability and can be used for cell detection by MRI.

## 2. Results

### 2.1. Encapsulin Expression

Prior to the experiment, hAD-MSCs were subjected to multipotency assay and flow cytometric analysis. The mesenchymal identity was confirmed by the cells’ potential for chondrogenic, osteogenic, and adipogenic differentiation. The CD profile of hAD-MSCs was revealed by flow cytometry (CD34−, CD44+, CD105+, CD73+, CD90+, CD117±) was typical for MSCs (Figure S1).

**Figure 1:**
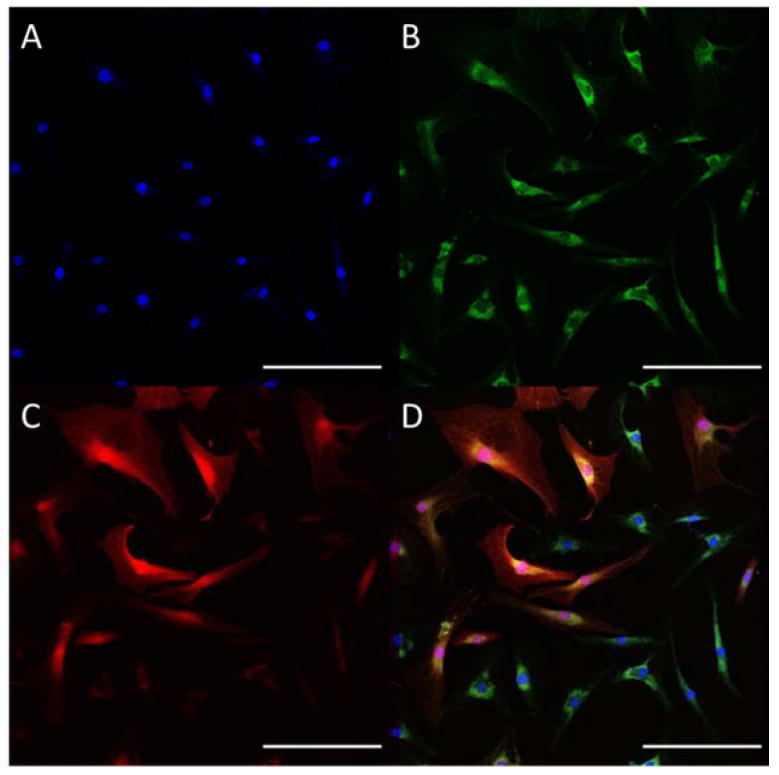
hAD-MSCs 48 h after infection by lentiviral vectors carrying genes encoding EncA-FLAG, EncBCD, and mZip14. Nuclei are stained with DAPI (**A**). Green channel (**B**) corresponds to GFP fluorescence; red channel (**C**) corresponds to RFP signal, (**D**)—merge image. Laser scanning confocal microscopy, scale bar 200 µm.

We used three lentiviral vectors to achieve stable expression of *Mx* encapsulin-encoding genes in MSCs: two of them had encapsulin protomer and cargo protein (EncA-FLAG and EncBCD), and the third vector had genes encoding iron transporter mZip14. Each vector also carried fluorescent protein genes (GFP for EncA-FLAG and RFP for EncBCD and mZip14). Laser confocal microscopy images of the stable cell line obtained after transduction (hAD-MSCs-Mx) presented in Figure 1 show either GFP or RFP fluorescence and double-positive cells.

**Figure 2:**
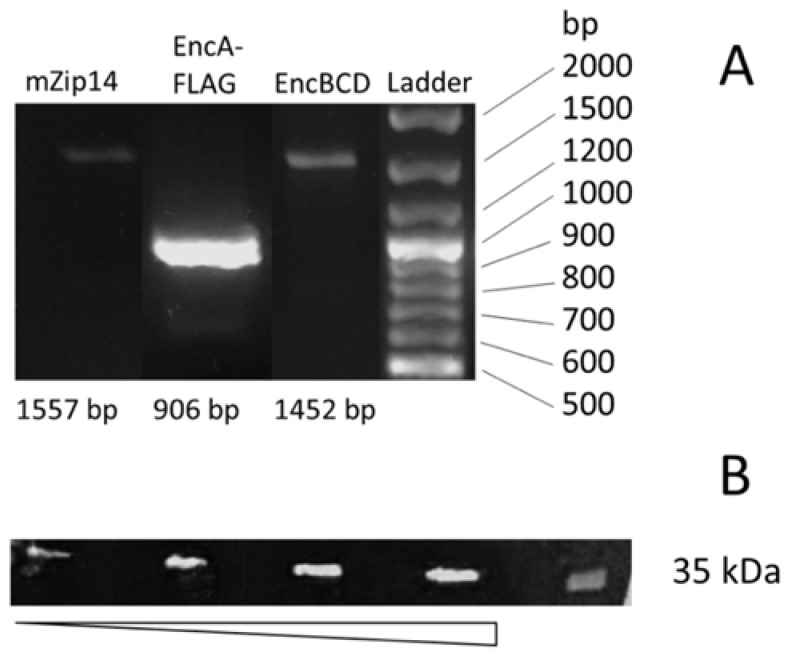
(**A**) Reverse transcription-polymerase chain reaction (RT-PCR) analysis of hAD-MSCs-Mx; (**B**) Western blot analysis against FLAG-tag on protomer proteins.

In this experiment, the fluorescent signal was not a direct indicator of transduction efficiency. It did not allow us to conclude that all three genes were integrated into the cell genome. To this end, we confirmed the expression of encapsulin genes by reverse transcription-polymerase chain reaction (RT-PCR, Figure 2A). Additionally, EncA expression in cells was verified by Western blot analysis against FLAG-tag on the protomer protein (Figure 2B). It clearly showed a single band with a molecular weight of around 35 kDa, and the signal level in Western blot increased with the amount of total protein loaded into the gel.

### 2.2. Iron Biomineralization

**Figure 3:**
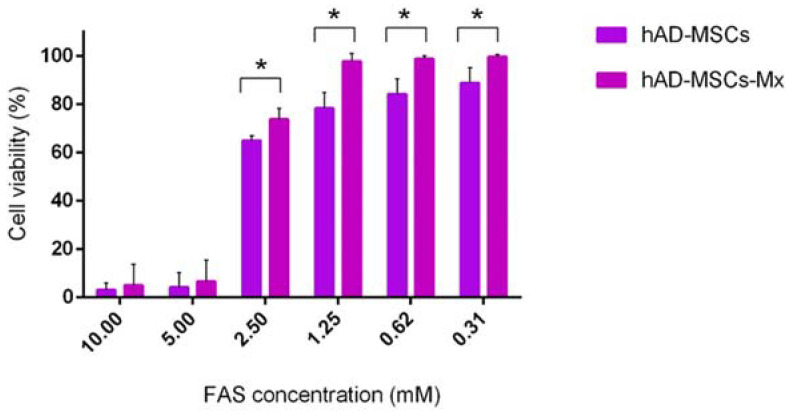
MTS assay evaluating the cytotoxicity of various concentrations of FAS in hAD-MSCs and hAD-MSCs-Mx. The data are shown as the mean + S.D. of three independent experiments; *p*-values were calculated using a one-tailed t-test, assuming unequal variances (* indicate *p*-values < 0.05).

After corroborating the expression of target *Mx* encapsulin genes in the stable hAD-MSCs-Mx cells and their supplementation with Fe, we tested the associated cytotoxic effects. Figure 3 shows the results of the MTS assay. The viability of hAD-MSCs-Mx cells after 24 h of incubation with FAS in a concentration range from 0.31 to 2.5 mM was higher than that of hAD-MSCs.

**Figure 4:**
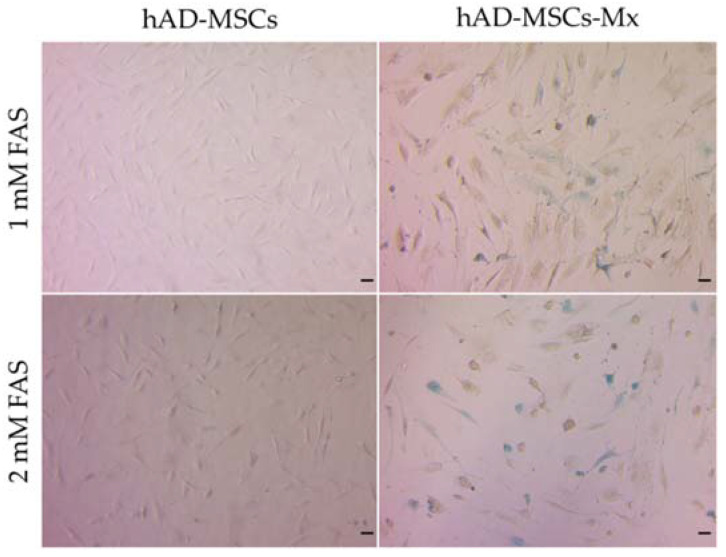
Prussian blue staining of hAD-MSCs and hAD-MSCs-Mx after 24 h incubation with 1 mM or 2 mM FAS. White-field microscopy, Zeiss Primo Vert, scale bar 50 µm.

After incubation with 1–2 mM FAS, Prussian blue staining of hAD-MSCs-Mx cells showed accumulated iron deposits in the cytoplasm and nucleus. Interestingly, non-transduced cells did not demonstrate such patterns (Figure 4).

**Figure 5:**
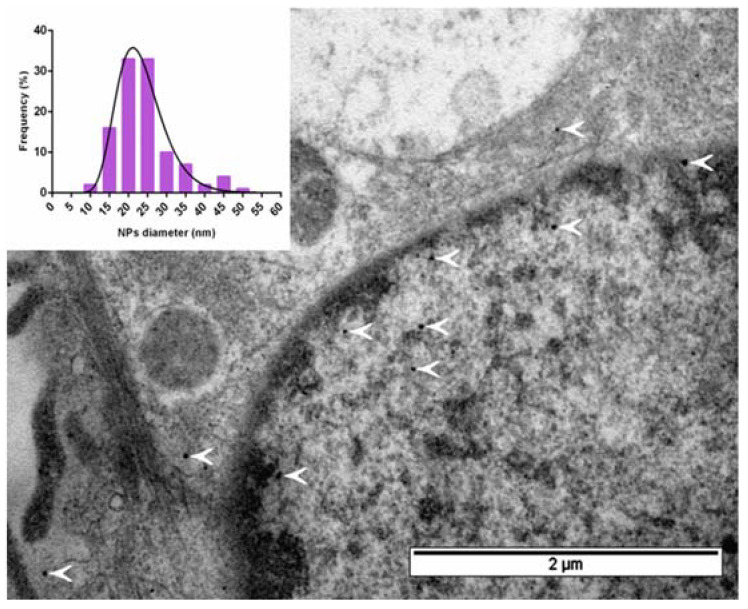
Bright-field TEM image of an ultrathin section of hAD-MSCs-Mx cells after 24 h of incubation with 2 mM FAS; white arrows indicate iron oxide deposits inside encapsulin nanocompartments. The inset illustrates the size distribution of iron oxide cores inside the encapsulin shell. Scale bar 2 µm.

Direct visualization of nanoparticles formed inside the encapsulin shell was performed by transmission electron microscopy (TEM, Figure 5). In TEM microphotographs, we detected electron-dense spherical nanoparticles located in both the cytoplasm and nucleus with an average diameter of 24 ± 8 nm, which correlated with the approximate diameter of the inner diameter of *Mx* encapsulin.

Finally, we evaluated the possibility of monitoring of hAD-MSCs-Mx via MRI. T2 relaxation times for hAD-MSCs-Mx (234 ± 18 ms) after 24 h incubation with 2 mM FAS were lower than for hAD-MSCs incubated with 2 mM FAS (545 ± 29 ms) (Supplementary Figure S2).

## 3. Discussion

In previous studies, heterologous expression of the *Mx* encapsulin system and the iron loading of nanocompartments were shown in mammalian HEK293T cells [33,35]. Moreover, the authors successfully demonstrated the possibility of MRI detection of such cells after their intracranial injection into the rat brain. In another study, encapsulin-based systems were expressed in human hepatocellular carcinoma cells HepG2 and visualized by MRI [34,35]. The authors hypothesized that this approach contributed to preclinical studies of tumor development and could potentially be used for genetically encoded tumor cell tracking. However, none of these studies described a possible direct application in clinically relevant techniques.

We chose human MSCs as a model cell line in our work due to the great interest and perspective of using MSCs in regenerative medicine. MSCs have the unique ability to self-renew and differentiate into other tissues of mesodermal origin such as bone, cartilage, adipose, muscle, and others. Furthermore, MSCs do not show a high immune response due to their unique phenotype, which allows them to escape recognition by cells of the immune system [36]. Two additional advantages of MSCs are easy isolation and cultivation, especially in comparison with induced pluripotent stem cells (iPSCs). Therefore, MSCs have already found many applications, both in clinical practice and in biomedical research. For example, in 2011, the Ministry of Food and Drug Safety of the Republic of Korea approved Cartistem^®^, a product for therapy of traumatic or degenerative osteoarthritis utilizing MSCs obtained from umbilical cord blood by Medipost [37]. Afterward, alternative products based on MSCs, including HeartiCellgram^®^, Mesoblast, TiGenix и Stempeutics, were approved for the treatment of various diseases by regulatory institutions all over the world. According to Clinicaltrials.gov, there are more than 300 clinical studies of therapies for different diseases using MSCs nowadays. MSCs are undergoing the second phase of clinical trials as a therapeutic drug to treat Acute Respiratory Distress Syndrome [38] and treat osteoarthritis of the knee [39]. Several clinical trials of MSCs for therapy of amyotrophic lateral sclerosis were completed [40,41].

In contrast to HEK293T cells, MSCs show dramatically lower transfection efficiency and cell viability after the transient transfection with lipofectamine or other reagents, as previously shown [42], thus requiring the use of viral vectors. To achieve stable expression of *Mx* encapsulin encoding genes in MSCs, we performed two sequential rounds of lentiviral transduction with a multiplicity of infection of 4 for each lentiviral vector and further selection on puromycin. Importantly, in [33,34,35], heterologous expression of encapsulins was achieved by the transient transfection, while in our study, the lentiviral transduction allowed us to obtain a stable cell line.

The co-expression of *Mx* encapsulin shell protomer (EncA) with ferroxidase cargo proteins (EncBCD) and an iron transporter (mZip14) in MSCs led to the formation of iron oxide crystals inside the encapsulin shells after the incubation of MSCs with FAS. First, we evaluated the effects of FAS on the viability of hAD-MSCs and hAD-MSCs-Mx. One of the established functions of encapsulin proteins is the protection of bacteria from reactive oxygen species (ROS) [32]. Iron ions catalyze the production of ROS by the Fenton reaction [43]. The sequestration of iron inside encapsulins in the form of iron oxide can reduce the total amount of free Fe^2+^/ Fe^3+^ ions, thus decreasing oxidative stress. We proposed that the same effect could be found in eukaryotic cells. The results of the MTS assay showed that the viability of hAD-MSCs-Mx was higher than hAD-MSCs after the incubation with the same concentration of FAS.

Prussian Blue staining, based on precipitation of blue-colored iron ferrocyanide, is among the common approaches to visualizing intracellular iron-containing structures such as iron oxide nanoparticles. We detected these precipitates in hAD-MSC-Mx cells but not in hAD-MSCs cells. This demonstrated that mZip14 (expressed in hAD-MSCs-Mx) was one of the crucial components required for proper iron transport from extracellular media followed by iron sequestration in the cells. Prussian blue staining also showed that the accumulation of intracellular iron deposits was dose-dependent. However, Prussian blue staining alone was insufficient to prove that iron was deposited inside the encapsulin nanocompartments. Instead, iron oxide crystals could only precipitate in the cell cytoplasm or on the cell membrane. To confirm the formation of iron oxide nanoparticles inside the encapsulins shells, we performed TEM imaging of the ultrathin sections of hAD-MSCs-Mx cells. TEM micrographs demonstrated the presence of electron-dense nanoparticles in the cell cytoplasm and nucleus with an average diameter of 24 ± 8 nm, nicely correlated with the diameter of the inner sphere of the encapsulin shell, with an outer diameter equal to 32 nm and inner diameter equal to 26 nm [32]. Therefore, we concluded that hAD-MSCs-Mx cells produced iron-containing nanoparticles encapsulated into *Mx* protein nanocompartments.

The intracellular formation of iron-containing nanoparticles itself does not guarantee their high contrast properties in MRI because the iron oxide can be present in a form with poor magnetic properties. Nevertheless, in our relaxometry measurement of cell pellets, the T2 relaxation time for hAD-MSCs-Mx (234 ± 18 ms) was significantly shorter than the T2 relaxation time for hAD-MSCs (545 ± 29 ms). In both cases, cells were measured in MRI after 24 h incubation with the 2 mM FAS. The cultivation of hAD-MSCs-Mx and their FAS supplementation still need to be optimized to isolate enough material and directly evaluate nanoparticles’ crystalline structures and their magnetic properties in the future.

Several studies have already proven the safety of using genetically modified MSCs in live systems. Such modified MSCs were successfully applied for the expression of therapeutic peptides in animal models; for example, E1A modified MSCs used in breast cancer therapy in mice [44]. In another study, Trx1-modified MCSs were used for cardiac function improvement in rat models of myocardial infarction [45]. MSCs were also used with interferon-γ expression for tumor growth suppression in mouse neuroblastoma and lung carcinoma models [46,47]. One possible issue experienced during translation into live systems would be the potential toxicity of 2 mM FAS as a source of Fe^2+^. On the other hand, some Fe^2+^ salts, for example, iron fumarate or lactate, have already been used in vivo [48]. These substances were applied in clinal practice for anemia treatment. Furthermore, there are mechanisms for maintaining the concentration of iron at a certain level in a living organism [49] which can also serve as a source of iron in encapsulins. Further work on increasing the encapsulin expression efficiency in hAD-MSCs and improving the sensitivity is required to allow long-term cell tracking by MRI in vivo.

## 4. Materials and Methods

### 4.1. Cell Culture

Samples of human adipose-derived MSCs, from the collection of the Cryobank of the Institute for Regenerative Medicine of Lomonosov Moscow State University (collection ID MSC_AD_MSU, www.human.depo.msu.ru, accessed on 1 October 2021), were used. Cells were cultured under standard conditions in a growth media (DMEM/F12) supplemented with 2 mM L-glutamine (Gibco) and 10% fetal calf serum (Gibco) and antibiotics (100 U/mL penicillin, 0.1 mg/mL streptomycin, Gibco) at 37 °C and 5% CO_2_ in T75 cultural flasks (Corning). Upon reaching 70–80% confluence, adherent hAD-MSCs were harvested by trypsinization and subcultured at a 1:3 ratio.

### 4.2. Flow Cytometry

Before cytofluorometry with FACS Aria III sorter (BD Biosciences), cells were labeled with primary antibodies to CD105, CD90, CD44, CD117, CD73, and CD34. Data were analyzed with BD FACS Diva 7 Software. Dead cells were stained with SytoxBlue Dead Cell stain (Invitrogen) and excluded from the analysis. Cell debris and doublets of cells were also excluded from analysis by direct and side light scattering parameters.

### 4.3. Differentiation of the Cells

To induce differentiation of MSCs to adipocytes, osteoblasts, and chondroblasts, the cells were cultured with StemPro^®^ Adipogenesis Differentiation Basal medium supplemented with StemPro^®^ Adipogenesis Supplement, StemPro^®^ Osteocyte/Chondrocyte Differentiation Basal medium in combination with StemPro^®^ Osteogenesis Differentiation Supplement or StemPro^®^ Osteocyte/Chondrocyte Differentiation Basal Medium in combination with StemPro^®^ Chondrogenesis Supplement, correspondingly (all reagents were from Gibco). To perform cell differentiation, they were cultured on a complete growth medium in 60 mm Petri dishes until they reached 50% confluency of the monolayer. Afterward, the growth medium was changed to the medium for differentiation. The medium for differentiation was replaced every 3 days; the differentiation experiments lasted for 21 days. When differentiation was finished, adipocytes, osteoblasts, and chondroblasts were stained with LipidTOX (Invitrogen), Alizarin Red (Sigma), and Alcian Blue (Sigma) dyes, respectively. Fluorescent EVOS FL (Life Technologies) and light inverted Primo Vert (Zeiss) microscopes were used to take pictures of stained cells.

### 4.4. Construction of Lentiviruses and Lentivirus Transduction of Cells

HEK293T cells were used for lentivirus particle production. The cells were seeded in 6-well plates covered with gelatin (5 × 10^5^ cells/well) and cultured in DMEM growth medium supplemented with 10% FBS, 2 mM L-glutamine, and antibiotics (50 units/mL penicillin, 50 ug/mL streptomycin) at 37 °C and 5% CO_2_. Transfection with Lipofectamine 3000 (Thermo Fisher, Waltham, MA, USA) was done according to the manufacturer’s instructions after 60–70% confluency of the cell monolayer was reached. The DNA composition was: plasmids with viruses’ genes Rev (19% by mass of total DNA), RRE (37% by mass of total DNA), VSS-G (7% by mass of total DNA), plasmids with genes of encapsulin system (pLCMV EncA-FLAG, pLCMV EncBCD) and genes of iron transporter pLCMV mZip14 (37% by mass of total DNA). Additionally, each vector included genes of fluorescent proteins (10% by mass of total transgene DNA). Individual virus vector was constructed for each of the components. Plasmids and P3000 reagent were mixed in an Opti-MEM medium. Lipofectamine in Opti-MEM was added to the solution (1:1), and the mixture was stirred and incubated for 15 min at room temperature. The mixture was then added to the cells. 24 h after transfection, the initial growth medium was replaced with DMEM supplemented with 2 mM L-glutamine, 2% FBS and antibiotics. Stocks of viruses were gathered after 48 and 72 h of cultivation: the growth medium was placed in 15 mL test tubes and centrifuged (500× *g*, 8 min); the supernatant was filtered with a syringe and 0.45 µm mesh filter. Transduction of the cells with the lentivirus vectors (constructed as described above) was done according to standard protocol in growth medium DMEM supplemented with 10% heat-inactivated FBS and polybrene (8 µg/mL, Sigma-Aldrich, Burlington, MA, USA). The multiplicity of the infection coefficient was equal to 4 for each virus.

### 4.5. Laser Scanning Confocal Microscopy

Fluorescence confocal micrographs were captured with the Nikon Eclipse Ti2 microscope equipped with lasers and scanning systems, ThorLabs, Nikon Apo 25X/1,10 water immersion objective lens, and 405, 488, 561, 642 nm lasers. Scanning was performed using the ThorImageLS Software, and ImageJ2 Fiji was used to process the images.

### 4.6. PCR with Reverse Transcription

The transduced cells were cultured until 90% of monolayer confluency in cultural dishes T-25. Then, total RNA was extracted with the help of an Extract RNA reagent (Evrogen) according to the manufacturer’s protocol. The samples were evaluated for approximate quantity and purity with spectrophotometry. Then, cDNA was synthesized with the help of Invitrogen SuperScript III Reverse Transcriptase (Thermo Fisher) and oligo-DT and random primers. The samples of cDNA and negative control (RNA sample without reverse transcriptase added) were used for classical PCR with Taq polymerase (Fermentas) and corresponding buffer. PCR products were separated with electrophoresis in 1% agarose gel. The desired fragments were identified by their length.

### 4.7. Western Blot Analysis

The cells were lysed with RIPA buffer, and the lysates were centrifuged (15 min, 14,000× *g*). Buffer for probes (5×) was added to 2, 5, 10, and 20 µL of cell lysate, and the samples were heated to 95 °C for 5 min and then cooled down by placing on ice. The samples were put in the gel, and electrophoresis was run on 80 V for 25 min and 100 V for 90 min. PageRuler-prestained protein ladder (Thermo Fisher) served as a length marker. The gel was placed to transfer buffer (25 mM Tris buffer, 192 mM glycine, pH 8.3, 10% ethanol, 0.25% SDS). Nitrocellulose membrane activated with 96% ethanol was placed over the gel. The samples transfer was performed in a bath with transfer buffer on 100 V for 1 h. Afterward, the membrane was washed 3 times from residues of transfer buffer in PBS-0.1% Tween (PBST). To block unspecific protein binding, the membrane was incubated in PBST supplemented with 5% skimmed milk for 2 h, then washed again. The membrane was incubated with primary antibodies against FLAG-tag (Sigma-Aldrich, 1:1000) for 2 h and then washed 3 times. Secondary goat anti-mouse IgG antibodies (Santa Cruz Biotechnology, 1:1000) conjugated with alkaline horseradish peroxidase. Clarity Max Western ECL Substrate kit (BioRad) was used to reveal the result according to the manufacturer’s protocol. The result was registered with the ChemidocMP Imaging system (BioRad).

### 4.8. Cytotoxicity Study of FAS In Vitro

To evaluate the potential cytotoxicity of FAS, hAD-MSCs and hAD-MSCs-Mx cells were seeded into 96-well plates (100 µL of growth medium with 1 × 10^4^ cells/well). Then, after 24 h, FAS was added to the cells in various concentrations (10, 5, 2.5, 1.25, 0.62 and и 0.31 mM). After 24 h incubation, the cells were washed with PBS, and a fresh growth medium with MTS-reagent was added (100 µL of growth medium and 20 µL of MTS-reagent in each well). Cells without FAS were used as a positive control. The cells were incubated with MTS-reagent for 4 h at 37 °C and 5% CO_2_ in the humid atmosphere. The test was done in three replicates. Optical density was measured with a Multiscan GO plate reader (Thermo Scientific), λ = 490 nm. Cell viability was calculated as:

Cell viability (%) = (A_s_ − A_b_)/(A_c_ − A_b_) × 100 (1)

where A_s_—mean optical density in sample wells, A_b_—mean optical density in blank wells, A_c_—mean optical density in positive control wells.

### 4.9. Prussian Blue Staining

To visualize the iron deposits inside the encapsulins, Prussian blue staining was performed. For this purpose, hAD-MSCs and hAD-MSCs-Mx cells were seeded in 24-well cell culture plates (3 × 10^4^ cells per well) and left overnight to attach to the plastic. FAS was diluted in deionized water and then in cell culture medium to obtain desired concentrations (1 mM and 2 mM) and added to the cells. After 24 h of incubation, hAD-MSCs and hAD-MSCs-Mx were washed with PBS and fixed with 4% paraformaldehyde. Subsequently, the cells were washed with deionized water and stained using Iron Staining Kit HT20 (Sigma-Aldrich). Afterward, the cells were washed again with deionized water, and photographs were taken using a light microscope Primo Vert (Zeiss).

### 4.10. TEM

hAD-MSCs-Mx were incubated in a growth medium (see above) supplemented with 2 mM FAS for 24 h and then fixed with 2% paraformaldehyde and 2.5% glutaraldehyde (Sigma-Aldrich) in PBS (pH 7.4) for 30 min. Then, the fixed cells were washed three times with PBS and postfixed with 1% osmium tetroxide for 1 h, RT. Afterward, the fixed cells were dehydrated in uprising ethanol series (50%, 70%, 80%, 95%) and embedded in an Epoxy medium (Sigma Aldrich) according to the manufacturer’s protocol. Ultrathin 70 nm sections were cut using Leica EM UC6 (Leica). TEM microphotographs were taken with a JEOL JEM 1400 microscope. Mean diameters were calculated for about 100 nanoparticles in ImageJ software, with nonlinear approximation fitting using log-normal distribution in GraphPad Prism.

### 4.11. MRI

For T2 relaxometry measurement, hAD-MSCs and hAD-MSCs-Mx cells were incubated with 2 mM FAS for 24 h. Afterward, cells were washed 3 times with DPBS, detached with TripLE, and centrifuged at 500× *g* for 4 min. The pellets (1.2 × 10^6^ cells each) were resuspended in 200 µL DPBS and transferred to 500 µL PCR tubes. Cells were then spun down at 500 g for 2 min and used for MRI. MRI images were acquired on ClinScan 7T system (Bruker Biospin, USA) in Spin Echo sequence with the following parameters: TR = 10,000, slice thickness 1.2 mm, FoV 84 × 120, base resolution 448 × 640, TE 8, 16, 24,…, 256.

## 5. Conclusions

In this work, we described human MSCs cell culture with a stable expression of the *Mx* encapsulin system for the first time. We showed a dose-dependent formation of iron oxide particles inside the encapsulin nanocompartments by TEM and Prussian blue staining, which increased the contrast in the T2-weighted MR images in vitro. Moreover, genetically encoded encapsulin labels did not affect the proliferation and viability of human MSCs and created a protective effect against a high concentration of iron ions. Therefore, this study is one more step toward new technology for genetically encoded in vivo stem cell tracking by MRI.

## Data Availability

All important data is included in the manuscript.

## Supplementary Materials

The following are available online at https://www.mdpi.com/article/10.3390/ijms222212275/s1.
