# Supplementary material for: Encapsulin Based Self-Assembling Iron-Containing Protein Nanoparticles for Stem Cells MRI Visualization
Source: Int J Mol Sci. 2021 Nov 12;22(22):12275. doi: 10.3390/ijms222212275 (PMC8618560; doi:10.3390/ijms222212275)
Supplement: Supplementary file 1 [file ijms-22-12275-s001.zip › ijms-1438818-supplementary.pdf]

International Journal of
Molecular Sciences

Article

Supplementary materials: Encapsulin based self-assembling iron-containing protein nanoparticles for stem cells MRI visualization

Anna N. Gabashvili, Stepan S. Vodopyanov, Nelly S. Chmelyuk, Viktoria A. Sarkisova, Konstantin A. Fedotov, Maria V. Efremova and Maxim A. Abakumov

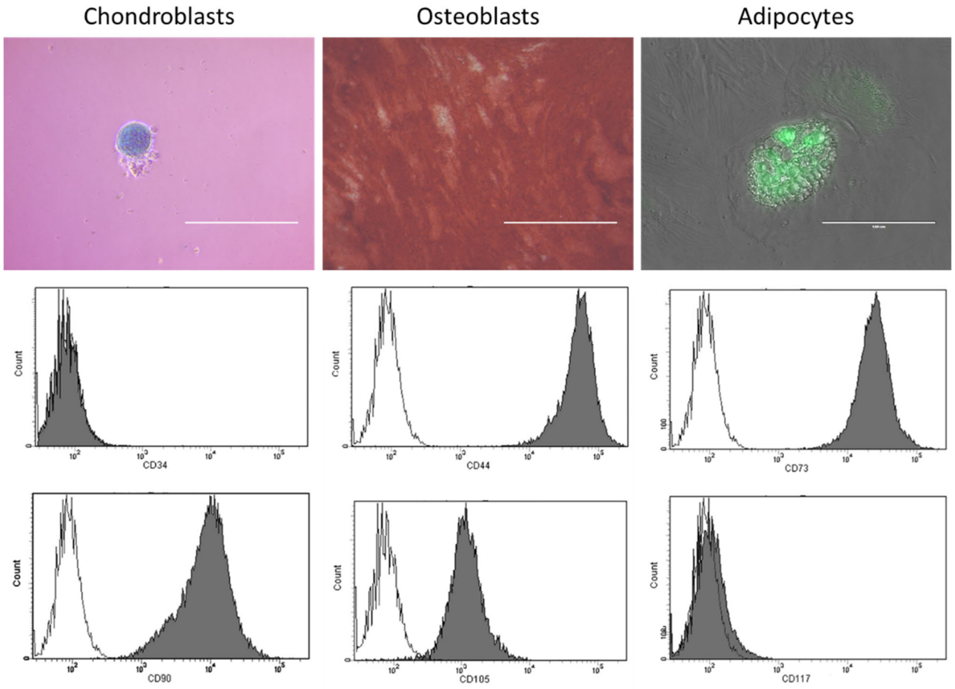

Figure S1. Staining of chondroblasts (Alcian Blue), osteoblasts (Alizarin Red) and adipocytes (LipidTOX). White field microscopy, Zeiss Primo Vert, scale bar 100 μm and fluorescence microscopy, Evose, scale bar 100 μm. Flow cytometry cells were labelled with primary antibodies to CD105, CD90, CD44, CD117, CD73 and CD34.

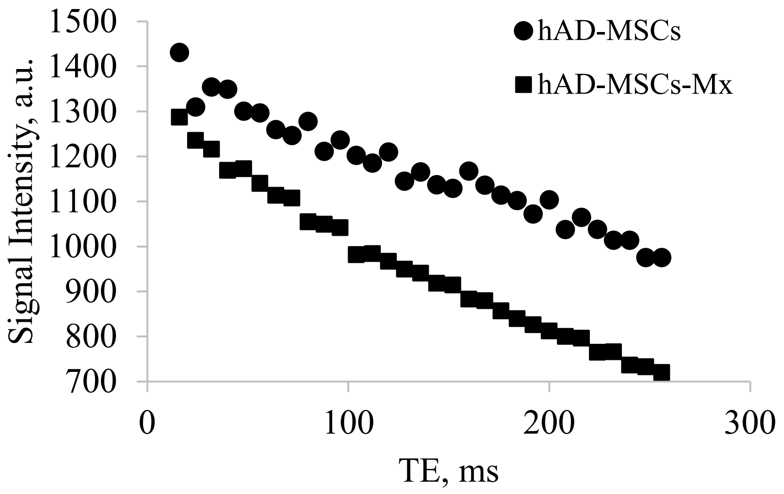

Figure S2. Signal intensity dependence on TE of cell pellets, obtained from hAD-MSCs and hAD-MSCs-Mx cells.
